# The Rothman Index predicts unplanned readmissions to intensive care associated with increased mortality and hospital length of stay: a propensity-matched cohort study

**DOI:** 10.1186/s13037-024-00391-2

**Published:** 2024-03-07

**Authors:** Philip F. Stahel, Kathy W. Belk, Samantha J. McInnis, Kathryn Holland, Roy Nanz, Joseph Beals, Jaclyn Gosnell, Olufunmilayo Ogundele, Katherine S. Mastriani

**Affiliations:** 1 Department of Surgery, East Carolina University, Brody School of Medicine, 27834 Greenville, NC USA; 2 Rocky Vista University, College of Osteopathic Medicine, 80134 Parker, CO USA; 3 Mission Health, HCA Healthcare North Carolina Division, 28803 Asheville, NC USA; 4 Spacelabs Healthcare, 98065 Snoqualmie, WA USA

**Keywords:** Rothman Index, Predictive analytics, ICU readmission, Mortality, Hospital length of stay, Patient safety

## Abstract

**Background:**

Patients with unplanned readmissions to the intensive care unit (ICU) are at high risk of preventable adverse events. The Rothman Index represents an objective real-time grading system of a patient’s clinical condition and a predictive tool of clinical deterioration over time. This study was designed to test the hypothesis that the Rothman Index represents a sensitive predictor of unanticipated ICU readmissions.

**Methods:**

A retrospective propensity-matched cohort study was performed at a tertiary referral academic medical center in the United States from January 1, 2022, to December 31, 2022. Inclusion criteria were adult patients admitted to an ICU and readmitted within seven days of transfer to a lower level of care. The control group consisted of patients who were downgraded from ICU without a subsequent readmission. The primary outcome measure was in-hospital mortality or discharge to hospice for end-of-life care. Secondary outcome measures were overall hospital length of stay, ICU length of stay, and 30-day readmission rates. Propensity matching was used to control for differences between the study cohorts. Regression analyses were performed to determine independent risk factors of an unplanned readmission to ICU.

**Results:**

A total of 5,261 ICU patients met the inclusion criteria, of which 212 patients (4%) had an unanticipated readmission to the ICU within 7 days. The study cohort and control group were stratified by propensity matching into equal group sizes of *n* = 181. Lower Rothman Index scores (reflecting higher physiologic acuity) at the time of downgrade from the ICU were significantly associated with an unplanned readmission to the ICU (*p* < 0.0001). Patients readmitted to ICU had a lower mean Rothman Index score (*p* < 0.0001) and significantly increased rates of mortality (19.3% vs. 2.2%, *p* < 0.0001) and discharge to hospice (14.4% vs. 6.1%, *p* = 0.0073) compared to the control group of patients without ICU readmission. The overall length of ICU stay (mean 8.0 vs. 2.2 days, *p* < 0.0001) and total length of hospital stay (mean 15.8 vs. 7.3 days, *p* < 0.0001) were significantly increased in patients readmitted to ICU, compared to the control group.

**Conclusion:**

The Rothman Index represents a sensitive predictor of unanticipated readmissions to ICU, associated with a significantly increased mortality and overall ICU and hospital length of stay. The Rothman Index should be considered as a real-time objective measure for prediction of a safe downgrade from ICU to a lower level of care.

## Background

Patients readmitted to an intensive care unit (ICU) for clinical deterioration after transfer to a lower level of inpatient care are vulnerable to adverse events, increased hospital length of stay, and potentially preventable complications, including death [1–5]. The optimal clinical triggers to predict patients with an unplanned readmission to ICU remain a topic of ongoing research [6–10]. The recent advance of predictive analytics for improving patient safety provides new objective grading tools to determine safe patient downgrades from ICU [11–13]. The Rothman Index (RI) was developed as an objective grading system of a patient’s overall condition and as a predictive tool of clinical deterioration based on the change in RI scores over time (ΔRI) [14–16]. The RI score is automatically generated in real-time from 26 different variables, including vital signs, laboratory parameters, and clinical assessments from nursing documentation in the electronic health record (EHR) [14–16]. Previous studies have shown that the RI represents a sensitive predictor of clinical deterioration in cancer patients on oncological wards [17] and in hospitalized patients with COVID-19 [18]. Furthermore, the RI was shown to predict adverse events and unplanned 30-day readmissions in colorectal surgery patients [19] and post-discharge adverse events in patients undergoing orthopedic surgery and spine procedures [20–22]. The present study was designed to investigate the role of the RI in predicting unanticipated ICU readmissions and to correlate the readmissions with the risk of mortality and prolonged hospital length of stay at a large tertiary referral academic medical center in the United States.

## Methods

### Study design, setting, and population

**Fig. 1:**
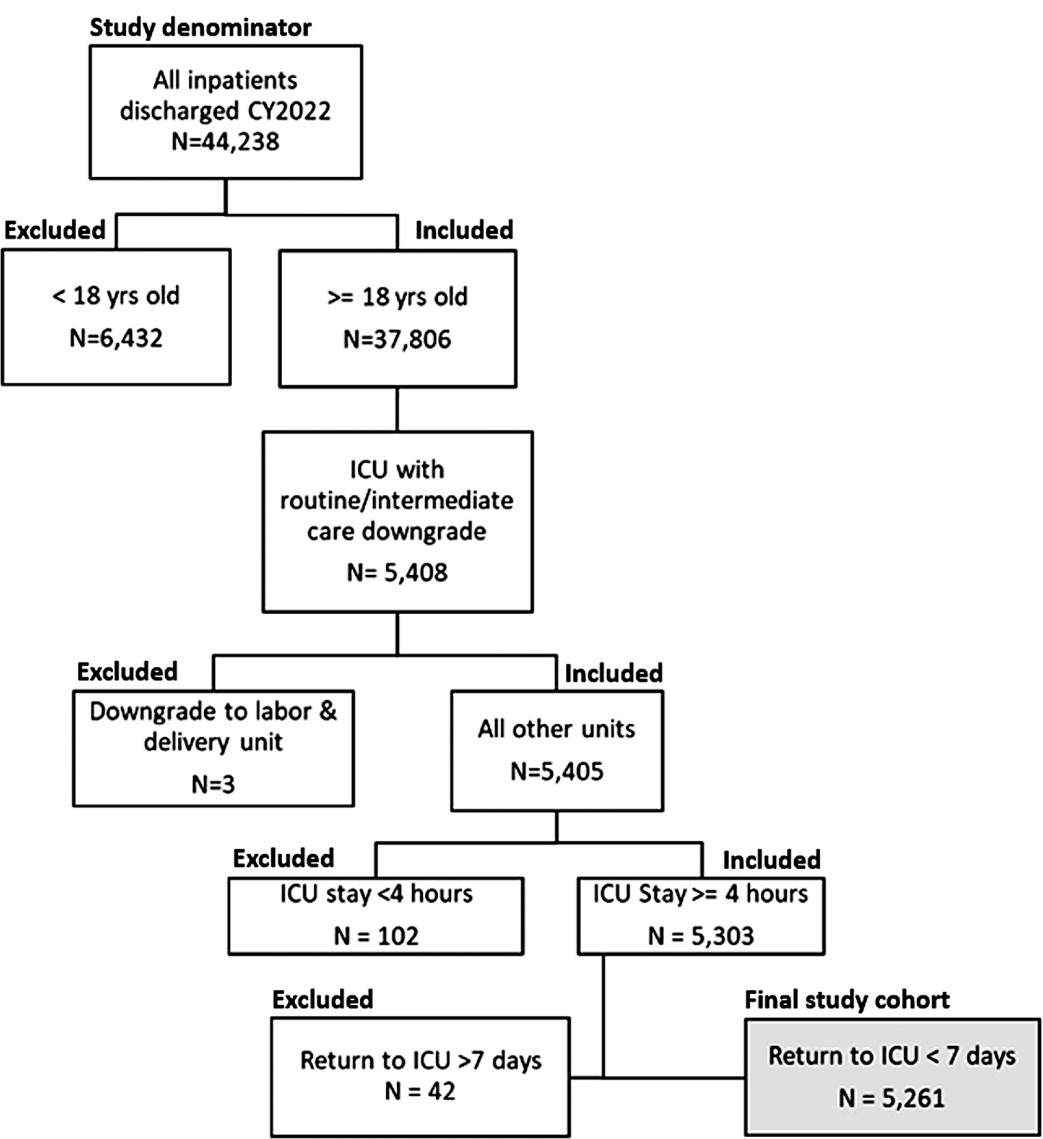
Patient selection flowchart

A retrospective propensity-matched cohort study was performed at a single tertiary referral academic medical center in Asheville, North Carolina (Mission Hospital / HCA Healthcare). Mission is an acute-care hospital with 853 licensed beds, including 147 ICU beds (87 adult / 60 pediatric/neonatal). The 87 adult ICU beds allocated to the care of patients included in this study comprise medical, surgical/trauma, cardiovascular, cardiothoracic surgery, neurological, and neurosurgical intensive care. The hospital has an affiliated ACGME-accredited residency and fellowship program which covers more than 170 trainees in 12 different training programs. The hospital’s primary service area comprises 18 counties in western North Carolina, providing the region’s only Level II trauma center, comprehensive stroke center, and children’s hospital. The study time-window was January 1, 2022, to December 31, 2022. Inclusion criteria consisted of all adult patients ≥ 18 years of age admitted to an ICU for a minimum of 4 h. The ICU admissions included medical or surgical indications for elective or urgent/emergent conditions. Exclusion criteria were patients < 18 years of age, downgrade to a labor/delivery unit, less than 4 h ICU length of stay, and a delayed readmission to ICU beyond 7 days. Analysis cohorts were identified based on the presence or absence of a return to the ICU during the inpatient stay. A return to the ICU was defined as a patient downgrade from the ICU to a routine or intermediate level of care setting with subsequent return to the ICU more than one hour and less than 7 days following the downgrade. The patient selection flowchart is depicted in Fig. 1.

This study was reviewed by the HCA Healthcare Institutional Review Board (IRB) and was deemed exempt from IRB oversight (ID# 2023 − 1146).

### The Rothman Index

The Rothman Index (Spacelabs Healthcare, Snoqualmie, WA, USA) is a real-time, composite measure of medical acuity for hospitalized patients which serves as a predictive analytics model designed to provide an objective measure for continuous monitoring of a patient’s clinical status and improvement or deterioration over time [14]. The RI is automatically generated in real-time and calculated by measuring deviation from a minimum risk value of the defined clinical variables, with a maximum score of 100 representing no deviation from minimum risk, and a deterioration in RI score reflecting deterioration in a patient’s clinical status. The RI was designed to be applicable to any patient with any underlying condition, independent of the specific diagnosis, type of treatment or intervention, and respective environment [14]. At Mission Hospital, the RI has been adopted as a tool with dual intent that allows (1) clinical monitoring and (2) appropriate decision-making for appropriate downgrades in the acuity level of care and safe discharge planning at daily multidisciplinary rounds (MDR).

**Fig. 2:**
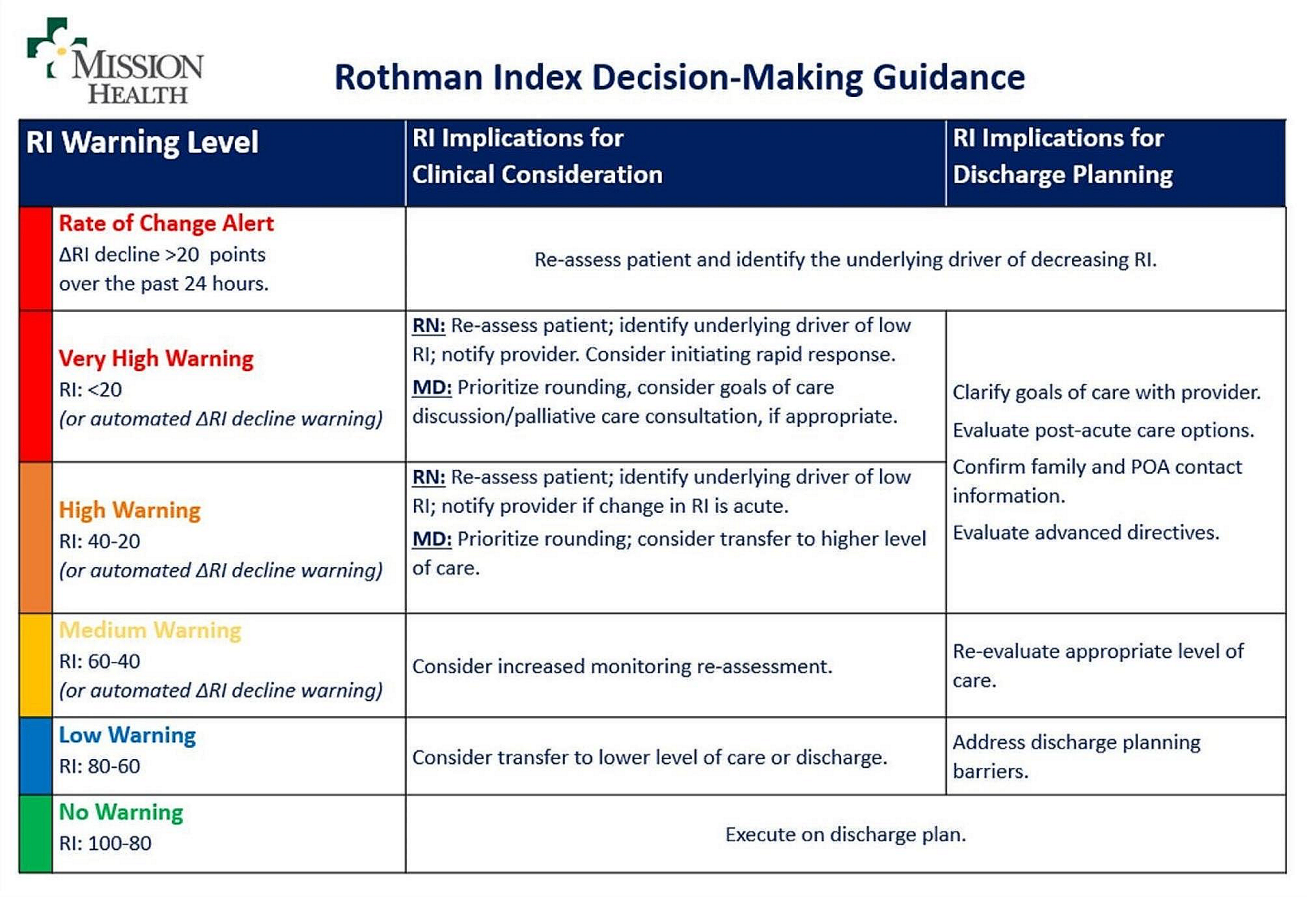
Rothman Index chart for clinical and patient downgrade/discharge decision-making at Mission Hospital (Asheville, North Carolina)

Figure 2 demonstrates the RI score grading thresholds pertaining to the respective decision-making recommendations used by clinical staff at Mission Hospital.

### Outcome measures

The primary outcome measure was in-hospital mortality or discharge to hospice for end-of-life care. Secondary outcome measures included overall hospital length of stay, ICU length of stay, and 30-day readmission rates. In-hospital mortality and discharge to hospice were defined using administrative discharge status codes. Cerner EHR Admission, Discharge and Transfer (ADT) system data were used to calculate both overall and ICU-specific length of stay. Hospital length of stay was defined as the number of days from admission to discharge, calculated to the hour. Length of ICU stay was calculated based on entry and exit date/times from the ICU. Readmissions were identified as any inpatient hospital visit to the same facility more than 6 h and less than 30 days from the time of inpatient discharge. Readmission analyses excluded patients with a discharge status of expired or discharged to hospice.

### Statistical analysis

Propensity matching was used to control for differences across cohorts to better estimate the impact of ICU returns on the primary and secondary outcomes. Matching covariates included features which could influence either return to ICU or the primary and secondary outcomes, specifically: patient age, gender, and admission type, medical/surgical classification for hospitalization using MS-DRG grouping, the first RI score during the visit, and the Charlson Comorbidity Index. The Charlson Comorbidity Index uses patient comorbidities to predict long-term mortality and is a commonly used algorithm to assess chronic conditions in hospitalized patients [23]. Logistic regression techniques were used to identify the cumulative probability of a return to ICU using the matching covariates. Cases of patients returning to the ICU were then matched to controls based on these probabilities using a 1:1 Greedy propensity matching algorithm and requiring at least a four decimal place match between the case and control. This algorithm attempts to match cases with the highest precision match first and continues to perform matches until no additional matches are found thereby minimizing the number of incomplete and inexact matches. Baseline demographics including patient characteristics (e.g., age, gender, race, ethnicity), visit characteristics (e.g., admission type, discharge status), and clinical features representing clinical status (e.g., first RI score, Charlson Comorbidity Index, medical vs. surgical care, COVID-19 diagnosis) were reported and compared across cohorts before and after the matching process. Counts and percentages were used to report and compare categorical outcomes including mortality, discharge to hospice and 30-day readmissions across cohorts while mean, median, and standard deviation were used to compare overall and ICU-specific length of stay. Chi-square tests were used to analyze differences between cohorts for categorical variables with Fisher’s exact test used for comparisons with small sample sizes. For continuous variables, ANOVA was used to analyze differences between cohorts with Mann-Whitney tests used for non-normal distributions. Multivariable regression models were used to estimate the impact of a return to the ICU on primary and secondary outcomes. Logistic regression models were used for in-hospital mortality, discharge to hospice, and readmission. General linear regression models with negative binomial distributions were used to evaluate length of stay overall and in the ICU. Model confounders included patient and visit characteristics, patient comorbidities, and clinical features indicating severity of illness and physiological status. All statistical tests were conducted using SAS version 9.4 (SAS Institute, Cary, NC).

A *p*-value < 0.05 was considered statistically significant.

### Sensitivity analysis

A sensitivity analysis evaluated the impact of time to ICU return on patient outcomes and hospital length of stay and to mitigate bias from the selection of a cut-point of seven days in the primary analysis. Outcomes were assessed and reported separately for returns to the ICU within three days and five days.

### Subgroup analysis

A descriptive analysis was conducted to evaluate the effectiveness of several features constructed from the RI to differentiate between patients with and without a return to the ICU. Analysis cohorts were identified based on the first ICU admission for both patients with and without a return to the ICU during the stay. ICU returns were defined using the methodology previously described. RI variables included the RI score at the time of the downgrade from the ICU to a lower level of care, the difference in the RI score between ICU entry and exit, the decrease in the RI score over the 24 h prior to downgrade and a binary indicator identifying if the patient was in a RI-generated warning at the time of downgrade. Configurable warnings based on either the RI score value or change in scores over time are part of the RI system functionality and serve an important role in operationalizing the RI for clinical decision support [24].

Propensity score matching with a 1:1 Greedy matching algorithm requiring at least a four decimal place match between the case and control was used to control for differences across cohorts similar to the primary analysis. For this subgroup analysis, matching variables included patient age, sex, type of ICU (i.e., medical vs. surgical/trauma), level of care in downgrade unit (i.e. routine vs. step-down), and first RI score in the ICU. A multivariable logistic regression model was used to evaluate the performance of the RI features on predicting returns to the ICU.

## Results

A total of 44,238 inpatients were admitted as inpatients to Mission Hospital during the one-year study time-window from January 1 to December 31, 2022. Of these, 5,261 ICU patients met the inclusion criteria for the study cohort of interest. A total of 212 patients (4.0%) had an unplanned readmission to the ICU within 7 days (Fig. 1). The two study cohorts with and without ICU readmission were stratified by propensity matching into equal group sizes of *n* = 181 each. Table 1 demonstrates the patient demographic data before and after the propensity matching process. Prior to matching, patients with readmission to ICU had a lower mean RI score (43.2 vs. 53.2, *p* < 0.0001) and a higher Charlson Comorbidity Index (3.2 vs. 2.2, *p* < 0.0001) at the time of hospital admission compared to the group of patients without a readmission to ICU. Patients with ICU returns also had significantly increased mortality in both the pre-match (18.9% vs. 2.2%, *p* < 0.0001) and post-match data (19.3% vs. 2.2%, *p* < 0.0001) as well as higher discharge to hospice transition rates in pre-match (14.6% vs. 3.8%, *p* < 0.0001) and post-match (14.4% vs. 6.1%, *p* = 0.0073) data. In addition, after matching the overall length of ICU stay (median 8.0 vs. 2.2 days, *p* < 0.0001) and total length of hospitalization (median 15.8 vs. 7.3 days, *p* < 0.0001) were significantly increased in patients readmitted to ICU compared to the non-readmission group (Table 2). There was no difference in overall 30-day readmission rates between the two study groups (*p* > 0.05). After controlling for patient characteristics and admission variables, ICU readmissions were associated with a more than 12-fold increase in the odds of a patient expiring during the hospitalization (OR = 12.71; 95% CI = 4.23–38.18; *p* < 0.001) and a more than 3-fold increase in the odds of discharge to hospice (OR = 3.44; 95% CI = 1.54–7.68; *p* = 0.0026). The odds ratios for all model covariates used in predicting mortality, hospice discharge and 30-day readmissions are shown in Table 3. Patients with an ICU return were predicted to have more than a 2 fold increase in length of stay (IRR = 2.19, 95% CI = 1.88–2.55, *p* < 0.0001) and 3 fold increase in ICU length of stay (IRR = 3.3, 95% CI = 2.77–3.95, *p* < 0.0001). Incident rate ratios of variables used in both models are shown in Table 4.

**Table 1:** Patient demographic data pre- and post- propensity match

	Pre- Propensity Match		Post- Propensity Match	
	No ICU Return	ICU Return	No ICU Return	ICU Return
Number of patients	5049	212	181	181
**Gender**				
Female	41.3%	38.2%	38.1%	40.3%
Male	58.6%	61.8%	61.9%	59.7%
Unknown	0.0%	0.0%	0.0%	0.0%
**Race**				
American Indian	1.4%	1.9%	0.0%	1.7%
African American	4.9%	3.8%	5.5%	3.3%
Other	1.1%	4.2%	0.6%	2.8%
Unknown	1.4%	2.4%	0.6%	2.8%
White/Caucasian	91.1%	87.7%	93.4%	89.5%
**Ethnicity**				
Hispanic/Latino	1.8%	1.4%	1.7%	1.1%
Non-Hispanic or Latino	90.9%	90.6%	90.6%	90.1%
Unknown	7.3%	8.0%	7.7%	8.8%
**Age at admission (years)**				
18–29	3.2%	1.9%	4.4%	2.2%
30–39	5.1%	2.4%	4.4%	2.2%
40–49	7.2%	7.1%	7.7%	7.2%
50–59	15.7%	17.9%	17.1%	17.1%
60–69	25.1%	32.5%	24.3%	32.6%
70–79	29.0%	27.4%	26.5%	27.6%
80–89	12.5%	8.5%	13.3%	8.3%
90+	2.2%	2.4%	2.2%	2.8%
Mean	64.8	64.9	64.4	64.9
**Admission type**				
Elective	25.8%	18.9%	19.9%	19.9%
Emergency	61.5%	68.4%	70.2%	66.3%
Urgent/Trauma	12.2%	12.3%	9.9%	13.8%
Unknown	0.4%	0.5%	0.0%	0.0%
**Discharge status**				
Against Medical Advice	1.2%	0.5%	2.2%	0.6%
Assisted Living	0.5%	0.5%	0.6%	0.6%
Expired	2.2%	18.9%	2.2%	19.3%
Home	49.2%	19.8%	44.2%	19.9%
Home Health	13.7%	9.0%	8.8%	8.8%
Hospice	3.8%	14.6%	6.1%	14.4%
Intermediate Care	0.2%	0.5%	0.0%	0.0%
Long Term Care	2.3%	9.4%	2.8%	9.9%
Other	0.6%	0.9%	0.0%	1.1%
Psychiatric Facility	1.2%	0.5%	1.7%	0.6%
Rehab	10.0%	7.1%	15.5%	7.7%
Skilled Nursing Facility	14.0%	14.6%	14.4%	14.4%
Interfacility Transfer	1.0%	3.8%	1.7%	2.8%
**Med/Surg MS-DRG**				
Information Missing	4.9%	1.4%	0.0%	0.0%
Medical	43.1%	41.5%	45.3%	42.5%
Surgical	52.0%	57.1%	54.7%	57.5%
**COVID-19 status**				
COVID-19 Positive	4.1%	7.1%	2.8%	7.7%
**Admission Rothman Index**				
< 20	8.0%	19.3%	13.8%	14.4%
20–29	8.7%	14.6%	12.2%	14.4%
30–39	14.2%	17.5%	16.6%	17.7%
40–49	14.5%	11.8%	16.6%	11.6%
50–59	13.0%	9.9%	11.0%	11.6%
60–69	12.8%	7.5%	9.9%	7.7%
70–79	12.1%	9.0%	11.0%	10.5%
80+	16.4%	10.4%	8.8%	12.2%
Information Missing	0.4%	0.0%	0.0%	0.0%
Mean	53.2	43.2	45.5	46.6
**Charlson Comorbidity Index**				
0	18.8%	15.1%	16.6%	17.7%
1	25.1%	13.7%	18.2%	14.9%
2	18.6%	15.6%	16.0%	16.6%
3	16.1%	17.9%	18.2%	18.8%
4	10.5%	14.6%	13.8%	13.8%
5	4.7%	8.5%	7.7%	8.3%
6	2.3%	4.7%	5.0%	4.4%
7	0.7%	3.8%	1.7%	2.8%
8	0.9%	0.0%	0.6%	0.0%
9	1.0%	1.4%	0.6%	1.1%
10	0.6%	1.9%	1.7%	0.6%
11	0.4%	1.4%	0.0%	1.1%
12	0.2%	0.5%	0.0%	0.0%
13	0.0%	0.5%	0.0%	0.0%
14	0.0%	0.0%	0.0%	0.0%
15	0.0%	0.5%	0.0%	0.0%
Mean	2.2	3.2	2.7	2.7

*Abbreviations* COVID-19, Coronavirus disease 2019; ICU, intensive care unit; MS-DRG, Medicare severity diagnosis-related groups

**Table 2:** Univariate analysis

	No ICU Readmission	ICU Readmission	p-value
Mortality Rate (%)	2.2%	19.3%	< 0.0001
Hospice Rate (%)	6.1%	14.4%	0.0073
30-Day Readmission Rate (%)	16.9%	25.0%	0.0873
Length of Stay (Days)			
Mean (SD)	10.5 (11.4)	23.2 (22.5)	
Median	7.3	15.8	< 0.0001
Initial ICU Length of Stay (Days)			
Mean (SD)	3.5 (4.7)	5.2 (6.0)	
Median	2.1	3.1	0.0019
Total ICU Length of Stay (Days)			
Mean (SD)	3.8 (5.2)	13.2 (18.6)	
Median	2.2	8.0	< 0.0001

*Abbreviations* ICU, intensive care unit; SD, standard deviation

**Table 3:** Logistic regression model for specific outcome measures

	Mortality	Hospice	30-Day Readmission
	OR (95% CI)	OR (95% CI)	OR (95% CI)
ICU Return vs. No ICU Return	12.707 (4.229–38.183)	3.440 (1.540–7.681)	1.656 (0.914–3.003)
Female vs. Male	0.799 (0.376–1.697)	1.167–0.559–2.435)	1.168 (0.632–2.160)
Urgent/Emergent vs. Elective Admission	2.675 (0.732–9.781)	1.732 (0.540–5.561)	0.713 (0.329–1.545)
Trauma vs. Elective Admission	2.348 (0.494–11.162)	0.752 (0.144–3.917)	0.520 (0.158–1.708)
Surgical vs. Medical Admission	0.431 (0.192–0.970)	0.493 (0.214–1.135)	0.783 (0.381–1.608)
First RI Score	0.988 (0.970–1.005)	0.971 (0.952–0.991)	0.999 (0.986–1.013)
Age (Years)	1.034 (1.004–1.064)	1.022 (0.993–1.051)	0.981 (0.960–1.002)
COVID-19 Positive	1.733 (0.512–5.860)	0.653 (0.133–3.209)	1.059 (0.263–4.262)
Charlson Comorbidity Index	1.057 (0.900–1.243)	1.165 (1.002–1.355)	1.157 (1.000–1.338)

*Abbreviations* CI, 95% confidence interval; COVID-19, Coronavirus disease 2019; ICU, intensive care unit; OR, odds ratio; RI, Rothman Index

**Table 4:** Negative binomial regression model for hospital LOS and ICU LOS

	Overall Length of Stay	ICU Length of Stay
	IRR (95% CI)	IRR (95% CI)
ICU Return vs. No ICU Return	2.187 (1.876–2.549)	3.307 (2.767–3.952)
Female vs. Male	0.946 (0.807–1.109)	1.013 (0.845–1.215)
Urgent/Emergent vs. Elective Admission	1.058 (0.865–1.293)	1.080 (0.854–1.366)
Trauma vs. Elective Admission	1.346 (1.019–1.778)	1.417 (1.033–1.945)
Surgical vs. Medical Admission	1.229 (1.039–1.455)	1.503 (1.237–1.826)
First RI Score	0.993 (0.989–0.996)	0.986 (0.982–0.990)
Age (Years)	0.989 (0.983–0.994)	0.991 (0.985–0.998)
COVID-19 Positive	1.254 (0.891–1.765)	1.640 (1.132–2.375)
Charlson Comorbidity Index	1.013 (0.977–1.051)	1.011 (0.970–1.054)

*Abbreviations* CI, 95% confidence interval; COVID-19, Coronavirus disease 2019; IRR, incident rate ratio; LOS, length of stay

Sensitivity analyses for 5-day and 3-day readmissions to ICU showed similar results to the 7-day ICU readmission analyses (Table 5). Specifically, the mortality rate was significantly increased in readmitted patients at either 5 days (19.8% vs. 2.4%, *p* < 0.0001) or 3 days (15.3% vs. 3.1%, *p* = 0.0006), compared to the cohort of patients without ICU readmission. In addition, both the hospital length of stay and the ICU length of stay were significantly increased among patients with a readmission to ICU either within 5 days (Hospital LOS: 15.4 vs. 8.4, *p* < 0.0001; ICU LOS: 7.9 vs. 2.8, *p* < 0.0001) or 3 days (Hospital LOS: 14.5 vs. 7.9, *p* < 0.0001; ICU LOS: 7.6 vs. 2.1, *p* < 0.0001).

**Table 5:** Outcome of 5-day and 3-day unplanned readmissions to ICU

		5-Day Returns to ICU				3-Day Returns to ICU		
		No ICU Return	ICU Return	P Value		No ICU Return	ICU Return	*P*-Value
Mortality Rate (%)		2.4%	19.8%	< 0.0001		3.1%	15.3%	0.0006
Hospice Rate (%)		7.8%	13.8%	0.0777		4.6%	13.0%	0.0163
30-Day Readmission Rate (%)		14.0%	22.5%	0.074		19.0%	24.5%	0.3329
**Length of Stay (Days)**								
Mean (SD)		11.8 (12.6)	21.5 (19.9)			11.1 (13.1)	21.7 (21.5)	
Median		8.4	15.4	< 0.0001		7.9	14.5	< 0.0001
**ICU Length of Stay (Days)**								
Mean (SD)		4.4 (5.3)	11.9 (16.2)			4.1 (8.9)	12.9 (19.3)	
Median		2.8	7.9	< 0.0001		2.1	7.6	< 0.0001

*Abbreviations* ICU, intensive care unit; SD, standard deviation

In the subgroup analysis, multivariate logistic regression was applied to determine significant predictors of an unanticipated readmission to ICU. Decreased odds of an unplanned ICU readmission were observed in patients who showed an increase in RI score, as a surrogate of improving clinical condition over time, from ICU admission until downgrade (OR = 0.975; 95% CI = 0.960–0.990, *p* = 0.0013) and within the last 24 h prior to downgrade to a lower level of care (OR = 0.945; 95% CI = 0.919–0.971, *p* < 0.0001). In addition, a higher Charlson Comorbidity Index was associated with increased odds of an unplanned readmission to the ICU (OR = 1.285; 95% CI = 1.141–1.448, *p* < 0.0001). The odds ratios for all covariates are shown in Table 6.

**Table 6:** Logistic regression analysis for predicting unplanned readmission to ICU within seven days

Parameter		OR (95% CI)	P-Value	
Female vs. Male		1.558 (0.936–2.594)	0.0879	
Patient age (years)		0.987 (0.969–1.005)	0.1472	
Surgical/trauma ICU vs. Medical ICU		0.760 (0.380–1.520)	0.4379	
Surgical vs. Medical Admissions		1.643 (0.773–3.493)	0.1972	
Downgrade Unit: Progressive Care vs. Med/Surg		1.139 (0.671–1.936)	0.6297	
COVID-19 Positive		2.121 (0.742–6.063)	0.1605	
Charlson Comorbidity Score		1.285 (1.141–1.448)*	< 0.0001	
RI Score at ICU Discharge		0.992 (0.976–1.008)	0.3030	
Difference RI Score ICU Admit to Discharge		0.975 (0.960–0.990)*	0.0013	
Decrease in RI score 24 h prior to ICU Discharge		0.945 (0.919–0.971)*	< 0.0001	
Downgrade to lower level of care in RI warning		1.393 (0.830–2.338)	0.2101	

*Abbreviations* COVID-19, Coronavirus disease 2019; ICU, intensive care unit; RI, Rothman Index

## Discussion

This retrospective observational cohort study on 5,261 critical care patients admitted to a tertiary academic medical center in North Carolina demonstrated that utilizing the RI as a predictive analytics tool identified patients at risk of an unsafe downgrade from ICU to a lower level of care. A total of 212 patients (4%) had an unanticipated readmission to the ICU within 7 days. The unplanned readmissions were associated with a prolonged median ICU length of stay (8.0 vs. 2.2 days), overall hospital length of stay (15.8 vs. 7.3 days), and significantly increased odds of mortality (19.3% vs. 2.2%) compared to a propensity-matched control cohort without an unplanned readmission to ICU. The pertinent literature has previously shown that patients who are readmitted to intensive care have significantly higher mortality rates (21–40%) compared to patients discharged from ICU without a readmission (3.6–8.4%) [25]. Different screening tools have been previously investigated to determine which patients are safely downgraded from ICU to a lower level of care. The application of the “National Early Warning Score” (NEWS) as a risk stratification tool was shown to identify patients at high risk for deterioration after discharge from a surgical trauma ICU [25, 26]. These so-called “physiological aggregate weighted track and trigger systems” are designed to allocate specific thresholds derived from clinical vital signs (blood pressure, heart rate, respiratory rate) to define triggers for clinical responses and to facilitate the decision-making for the safety of downgrades to a lower level of care [27]. A review of the literature revealed that ICU readmission rates range from 1.5 to 13.4%, with the main underlying risk factors being male gender, pre-existing comorbidities, a comatose state (Glasgow Coma Scale score ≤ 8), and respiratory failure [25]. Impressively, the most frequently identified root cause of ICU readmission was respiratory failure, with patients requiring mechanical ventilation at time of the ICU return having a significant increased risk of mortality above 25% [28, 29]. The innovative aspect of the current study was to leverage predictive analytics using the RI which is a real-time “point-of-care” solution that is comprised of a broad range of discrete clinical inputs beyond vital signs, including laboratory parameters and clinical assessments from nursing documentation [14–16]. The RI was shown in previous studies to represent a sensitive predictor of clinical deterioration and unanticipated readmissions in patients with cancer, COVID-19, and postoperatively after colorectal, orthopedic, and spinal surgery [17–22]. The present study is the first, to our knowledge, to determine the predictive value of the RI for patients readmitted to a large multidisciplinary 87-bed ICU with regards to identifying patients at risk for an unplanned readmission associated with prolonged LOS and significantly increased mortality.

An important consideration when operationalizing any predictive tool in support of clinical care is ensuring effective integration into clinical workflow. Predictive tools that serve to provide additional insight to clinicians by augmenting, rather than competing with or replacing, clinical judgment stand to add the greatest value and achieve the widest adoption. Additionally, such models should only be incorporated into the workflow when accompanied by a clear understanding of situations in which the information may be relevant as well as how to interpret the information. At Mission Hospital in North Carolina, the RI is a component of clinical and multi-disciplinary rounds where it functions as an objective and widely understood reference point to aid discussions and decisions related to transitions in care, including proactive care escalation, safely downgrading patients to lower care levels, and readiness for discharge. In the authors’ experience, the clinical utility of the RI expands beyond determining the safety of ICU downgrades, to include rapid response alerts in patients with acute clinical deterioration (ΔRI warning), staffing decisions based on RI values reflecting the individual patient’s clinical acuity; and discharge planning considerations at multidisciplinary rounds (Fig. 2).

Based on this study we would propose incorporating a threshold RI score as well as declining trends in the RI since the time of ICU admission and within the most recent 24 h as part of a standardized transfer report or a multi-disciplinary rounding process to prompt clinical re-evaluation of patient readiness for downgrade. It is important as part of this process to understand not just the change in the RI score but also the underlying clinical drivers; factors such as changes in patient oxygen saturation or blood pressure, clinical observations of retractions or stridor in breath sounds, or newly observed signs of delirium.

The main shortcoming and limitation of this study is reflected by the retrospective study design which precludes the ability of determining the “true” sensitivity of the RI and ∆RI values in predicting unanticipated ICU readmissions in a prospective patient population. A further limitation of this study stems from the low rate of ICU returns spanning a diverse clinical population and etiology, precluding more granular diagnosis or condition sub-segmentation or propensity matching. However, the magnitude of the difference reported strongly suggests that ICU returns impact both mortality and LOS. Additional analyses on a larger volume of patients could provide further insight into the magnitude of the impact which ICU returns have on mortality and length of stay as a function of the reasons for readmission. In this regard, a strength of the RI in supporting downgrade decisions is that the RI is constructed as an overall measure of physiologic condition applicable across clinical conditions. A deeper analysis of RI cut-points and trends by specific etiology of ICU returns could help to refine relevant RI metrics and facilitate more effective clinical operationalization. Future prospective controlled studies will have to be designed to validate the insights from this single-center retrospective cohort study.

## Conclusion

The RI was shown to represent a sensitive predictor of unplanned readmissions to ICU at a large tertiary academic referral center in the United States. Patients who were readmitted after downgrade from ICU had a significantly increased mortality and overall hospital length of stay. Notably, the RI is intended to support clinical workflow and to provide guidance for informed decision making, and not to replace clinical judgment by physicians and nurses providing bedside care to their patients. The insights from the current study imply that the Rothman Index should be considered as a real-time objective measure of a patient’s clinical status for prediction of a safe downgrade from ICU to a lower level of inpatient care.

## Data Availability

No datasets were generated or analysed during the current study.

## References

[1] Moore D, Durie ML, Bampoe S, Buizen L, Darvall JN (2021). The risk of postoperative deterioration of non-cardiac surgery patients with ICU referral status who are admitted to the regular ward: a retrospective observational cohort study. Patient Saf Surg.

[2] Morgan M, Vernon T, Bradburn EH, Miller JA, Jammula S, Rogers FB (2020). A comprehensive review of the outcome for patients readmitted to the ICU following trauma and strategies to decrease readmission rates. J Intensive Care Med.

[3] Coughlin DG, Kumar MA, Patel NN, Hoffman RL, Kasner SE (2018). Preventing early bouncebacks to the neurointensive care unit: a retrospective analysis and quality improvement pilot. Neurocrit Care.

[4] Stahel PF, Mehler PS (2009). Medical emergency teams and rapid response triggers: the ongoing quest for the ‘perfect’ patient safety system. Crit Care.

[5] Alban RF, Nisim AA, Ho J, Nishi GK, Shabot MM (2006). Readmission to surgical intensive care increases severity-adjusted patient mortality. J Trauma.

[6] Bradburn EH, Jammula S, Horst MA, Morgan M, Vernon TM, Gross BW, Miller JA, Cook AD, Kim PK, Von Nieda D (2020). An analysis of outcomes and predictors of intensive care unit bouncebacks in a mature trauma system. J Trauma Acute Care Surg.

[7] Nathan CL, Stein L, George LJ, Young B, Fuller J, Gravina B, Dubendorf P, Kasner SE, Kumar MA (2022). Standardized transfer process for a neurointensive care unit and assessment of patient bounceback. Neurocrit Care.

[8] Manning SW, Orr SL, Mastriani KS (2020). General surgery residency and emergency general surgery service reduces readmission rates and length of stay in nonoperative small bowel obstruction. Am Surg.

[9] Blackburn HN, Clark MT, Moorman JR, Lake DE, Calland JF (2018). Identifying the low risk patient in surgical intensive and intermediate care units using continuous monitoring. Surgery.

[10] Christmas AB, Freeman E, Chisolm A, Fischer PE, Sachdev G, Jacobs DG, Sing RF (2014). Trauma intensive care unit ‘bouncebacks’: identifying risk factors for unexpected return admission to the intensive care unit. Am Surg.

[11] Alarhayem AQ, Muir MT, Jenkins DJ, Pruitt BA, Eastridge BJ, Purohit MP, Cestero RF (2019). Application of electronic medical record-derived analytics in critical care: Rothman Index predicts mortality and readmissions in surgical intensive care unit patients. J Trauma Acute Care Surg.

[12] Gotur DB, Masud F, Paranilam J, Zimmerman JL (2020). Analysis of Rothman Index data to predict postdischarge adverse events in a medical intensive care unit. J Intensive Care Med.

[13] Piper GL, Kaplan LJ, Maung AA, Lui FY, Barre K, Davis KA (2014). Using the Rothman index to predict early unplanned surgical intensive care unit readmissions. J Trauma Acute Care Surg.

[14] Rothman MJ, Rothman SI, Beals J (2013). Development and validation of a continuous measure of patient condition using the Electronic Medical Record. J Biomed Inf.

[15] Finlay GD, Rothman MJ, Smith RA (2014). Measuring the modified early warning score and the Rothman index: advantages of utilizing the electronic medical record in an early warning system. J Hosp Med.

[16] Wengerter BC, Pei KY, Asuzu D, Davis KA (2018). Rothman Index variability predicts clinical deterioration and rapid response activation. Am J Surg.

[17] Fitzpatrick N, Guck D, Van de Louw A (2018). Impact of Rothman index on delay of ICU transfer for hematology and oncology patients deteriorating in wards. Crit Care.

[18] Moguillansky D, Sharaf OM, Jin P, Samra R, Bryan J, Moguillansky NI, Lascano J, Kattan JN (2022). Evaluation of clinical predictors for major outcomes in patients hospitalized with COVID-19: the potential role of the Rothman Index. Cureus.

[19] Peterson KJ, O’Donnell CM, Eastwood DC, Szabo A, Hu KY, Ridolfi TJ, Ludwig KA, Peterson CY (2023). Evaluation of the Rothman Index in predicting readmission after colorectal resection. Am J Med Qual.

[20] Kleven AD, Middleton AH, Kesimoglu ZN, Slagel IC, Creager AE, Hanson R, Bozdag S, Edelstein AI (2022). Do in-hospital Rothman Index scores predict postdischarge adverse events and discharge location after total knee arthroplasty?. J Arthroplasty.

[21] McLynn RP, Ottesen TD, Ondeck NT, Cui JJ, Rubin LE, Grauer JN (2018). The Rothman Index is associated with postdischarge adverse events after hip fracture surgery in geriatric patients. Clin Orthop Relat Res.

[22] McLynn RP, Ondeck NT, Cui JJ, Swanson DR, Shultz BN, Bovonratwet P, Grauer JN (2018). The Rothman Index as a predictor of postdischarge adverse events after elective spine surgery. Spine J.

[23] Goellner Y, Tipton E, Verzino T, Weigand L (2022). Improving care quality through nurse-to-nurse consults and early warning system technology. Nurs Manag.

[24] Charlson ME, Pompei P, Ales KL, MacKenzie CR (1987). A new method of classifying prognostic comorbidity in longitudinal studies: development and validation. J Chronic Dis.

[25] McNeill H, Khairat S (2020). Impact of intensive care unit readmission on patient outcomes and the evaluation of the National Early warning score to prevent readmissions: literature review. MIR Perioper Med.

[26] Smith GB, Prytherch DR, Meredith P, Schmidt PE, Featherstone PI (2013). The ability of the National Early warning score (NEWS) to discriminate patients at risk of early cardiac arrest, unanticipated intensive care unit admission, and death. Resuscitation.

[27] Gao H, McDonnell A, Harrison DA, Moore T, Adam S, Daly K, Esmonde L, Goldhill DR, Parry GJ, Rashidian A, Subbe CP, Harvey S (2007). Systematic review and evaluation of physiological track and trigger warning systems for identifying at-rik patients on the ward. Intensive Care Med.

[28] Lee JY, Park SK, Kim HJ, Hong SB, Lim CM, Koh Y (2009). Outcome of early intensive care unit patients readmitted in the same hospitalization. J Crit Care.

[29] Sheetrit E, Brief M, Elisha O (2023). Predicting unplanned readmissions in the intensive care unit: a multimodal evaluation. Sci Rep.

